# Shape of a Common Protein Module Suggests Role as Molecular Switch

**DOI:** 10.1371/journal.pbio.0040221

**Published:** 2006-06-06

**Authors:** Mason Inman

A vital aspect of a neuron's job is deciding when to pass their cache of chemicals on to neighboring cells. To do this in a way that ensures effective communication, neurons must keep tight reins on their neurotransmitters—the chemical messengers they release to influence neighboring cells. Neurons quickly collect and then jettison these neurotransmitters, cycling through this process many times per second.

Now a new study reveals the structure of a fragment of Munc-13—a key protein in this process—showing how it could act as a switch that neurons use to toggle quickly between storing and releasing their neurotransmitters. For this switching role, the key part of the protein is one of its C _2_ domains (the C _2_A domain). This domain is widespread, suggesting that other proteins may function by a similar mechanism.

Neurons store their neurotransmitters in vesicles, tiny sacs that amass at the synapse between cells. There, they dock at the inside of the cell membrane and undergo one or more reactions that “prime” the vesicles to be ready to quickly unload their neurotransmitters outside the cell when the neuron fires. A cascade of protein interactions mediates these different steps.

These protein cascades not only allow neurons to do their usual duty, but they also help neurons process information. If neurons react quickly or sluggishly, reliably or not, to a neuron firing, this affects the signal that gets passed on to neighboring neurons. And, if their reactions change with time, this plasticity can help the animal remember, learn, and adapt.

To understand how the proteins interact, a key step is figuring out the shapes the proteins assume. Once they have the shapes, researchers can see how proteins, like pieces of a jigsaw puzzle, fit together. For example, this helps researchers make sense of chemical and biological data that suggest how proteins bind together and influence each other.

Josep Rizo and colleagues targeted a protein called Munc13-1 since its family of proteins plays important roles in preparing vesicles to release neurotransmitters, and Munc13-1 itself is known to have a role in the plasticity of synapses. Rizo and colleagues homed in on one of the key zones of Munc13-1, called the C _2_A domain. C _2_ domains are well known for being able to bind to phospholipids, the fatty acids that make up cell and vesicle membranes. But the researchers found an unexpected role for the Munc13-1 C _2_A domain: it can bind to other proteins as well.

To figure out the shape of Munc13-1's C _2_A domain, down to the atomic level, the researchers combined two methods: X-ray crystallography and nuclear magnetic resonance (NMR) spectroscopy. X-ray crystallography is the classic method for “solving” the mystery of a protein's shape. But not all proteins crystallize well, and so are not amenable to the method. Or if they do crystallize, their shape may be distorted from the form it normally takes when floating in a cell's watery interior.

Rizo and colleagues combined the two methods to create a detailed picture of the protein's shape. They used NMR spectroscopy to check how well the protein behaves in solution and to optimize the crystallization, aiding the X-ray studies. Also, data from the NMR spectroscopy on the shape of the protein helped them interpret the X-ray results. In the end, they were able to figure out the natural shape of the protein's C _2_A domain to atomic resolution.

These structural data also helped Rizo and colleagues make sense of their experiments designed to determine Munc13-1's binding partners. The researchers found that, in a pure solution, Munc13-1 proteins bound to each other in pairs. But when mixed with RIM2α (another protein that plays a key role in neurotransmitter release and neuron plasticity), then Munc13-1 formed pairs with RIM2α instead.

Combining the structural and binding data, the researchers were able to piece together a picture of how Munc13-1 could work as a switch. They found that the C _2_ domain of Munc13-1 has two adjacent and partially overlapping regions that can bind to other proteins. So two C _2_ domains could bind each other using one part of that domain, and another part of the domain could bind to RIM2α. But the C _2_ domain can't bind both at the same time. The researchers could see, once they had the shapes of the proteins, that they simply can't all fit together at once. Thus it seems that there is competition for Munc13-1 to bind either to itself or to other proteins. This mechanism could prepare the vesicles to be ready to unload the neurotransmitters when the neuron fires.

Since the C _2_ domain is widespread among proteins—and is highly similar among distantly related species—the findings could hold clues to understanding more roles for this common sequence of amino acids.

## 

**Figure:**
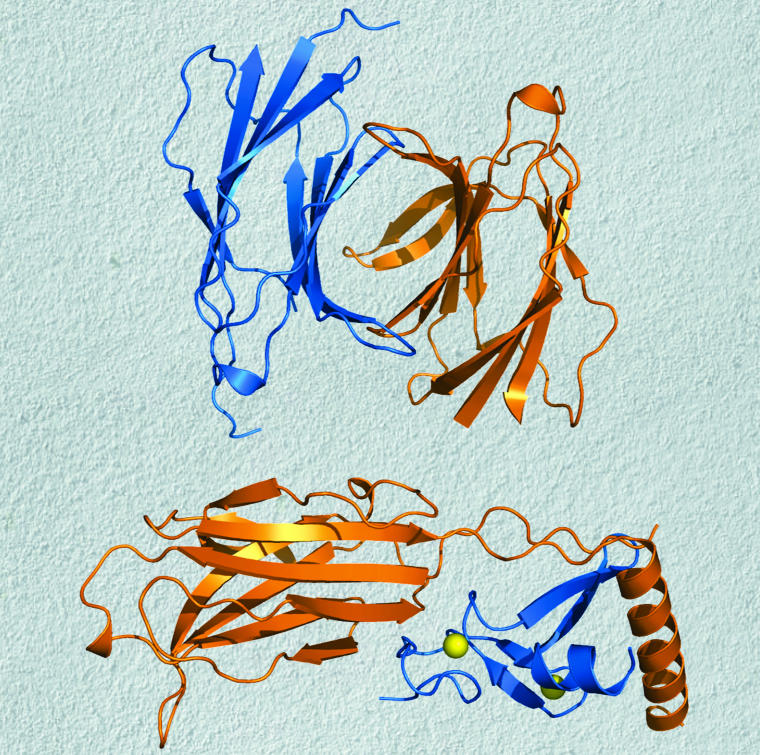
The crystal structures of a Munc13-1 homodimer (top) and a Munc13-1/RIM2α heterodimer (bottom) show how a C _2_ domain participates in two distinct protein–protein interactions that might couple synaptic vesicle priming to presynaptic plasticity.

